# Supplementary material for: Resolving the full spectrum of human genome variation using Linked-Reads
Source: Genome Res. 2019 Apr;29(4):635–45. doi: 10.1101/gr.234443.118 (PMC6442396; doi:10.1101/gr.234443.118)
Supplement: Supplemental Material [file supp_gr.234443.118_Supplemental_longranger-2.2.zip › longranger-2.2.tar.bz2!/lr2.2-public/lib/stan/cmdstan/stan/doc/api/html/containers__conversion_8hpp_source.html]

Stan: src/stan/math/matrix/containers\_conversion.hpp Source File


|  |  |
| --- | --- |
|  | Stan  2.5.0  probability, sampling & optimization |


- Main Page
- Related Pages
- Namespaces
- Classes
- Files

- File List
- File Members

All Classes Namespaces Files Functions Variables Typedefs Enumerations Enumerator Friends Macros Pages

- src
- stan
- math
- matrix

containers\_conversion.hpp

Go to the documentation of this file.

1 #ifndef STAN\_\_MATH\_\_MATRIX\_\_CONTAINERS\_CONVERSION\_HPP

2 #define STAN\_\_MATH\_\_MATRIX\_\_CONTAINERS\_CONVERSION\_HPP

3

4 #include <stan/math/matrix/Eigen.hpp>

5 #include <stan/meta/traits.hpp> //stan::scalar\_type

6 #include <vector>

7

8 namespace stan {

9  namespace math {

10

11  using Eigen::Dynamic;

12  using Eigen::Matrix;

13  using std::vector;

14

15  //matrix to\_matrix(matrix)

16  //matrix to\_matrix(vector)

17  //matrix to\_matrix(row\_vector)

18  template <typename T, int R, int C>

19  inline Matrix<T, Dynamic, Dynamic>

20  to\_matrix(Matrix<T, R, C> matrix) {

21  return matrix;

22  }

23

24  //matrix to\_matrix(real[,])

25  template <typename T>

26  inline Matrix<T, Dynamic, Dynamic>

27  to\_matrix(const vector< vector<T> > & vec) {

28  size\_t R = vec.size();

29  if (R != 0) {

30  size\_t C = vec[0].size();

31  Matrix<T, Dynamic, Dynamic> result(R, C);

32  T\* datap = result.data();

33  for (size\_t i=0, ij=0; i < C; i++)

34  for (size\_t j=0; j < R; j++, ij++)

35  datap[ij] = vec[j][i];

36  return result;

37  } else {

38  return Matrix<T, Dynamic, Dynamic> (0, 0);

39  }

40  }

41

42  //matrix to\_matrix(int[,])

43  inline Matrix<double, Dynamic, Dynamic>

44  to\_matrix(const vector< vector<int> > & vec) {

45  size\_t R = vec.size();

46  if (R != 0) {

47  size\_t C = vec[0].size();

48  Matrix<double, Dynamic, Dynamic> result(R, C);

49  double\* datap = result.data();

50  for (size\_t i=0, ij=0; i < C; i++)

51  for (size\_t j=0; j < R; j++, ij++)

52  datap[ij] = vec[j][i];

53  return result;

54  } else {

55  return Matrix<double, Dynamic, Dynamic> (0, 0);

56  }

57  }

58

59  //vector to\_vector(matrix)

60  //vector to\_vector(row\_vector)

61  //vector to\_vector(vector)

62  template <typename T, int R, int C>

63  inline Matrix<T, Dynamic, 1>

64  to\_vector(const Matrix<T, R, C>& matrix) {

65  return Matrix<T, Dynamic, 1>::Map(matrix.data(), matrix.rows()\*matrix.cols());

66  }

67

68  //vector to\_vector(real[])

69  template <typename T>

70  inline Matrix<T, Dynamic, 1>

71  to\_vector(const vector<T> & vec) {

72  return Matrix<T, Dynamic, 1>::Map(vec.data(), vec.size());

73  }

74

75  //vector to\_vector(int[])

76  inline Matrix<double, Dynamic, 1>

77  to\_vector(const vector<int> & vec) {

78  int R = vec.size();

79  Matrix<double, Dynamic, 1> result(R);

80  double\* datap = result.data();

81  for (int i=0; i < R; i++)

82  datap[i] = vec[i];

83  return result;

84  }

85

86  //row\_vector to\_row\_vector(matrix)

87  //row\_vector to\_row\_vector(vector)

88  //row\_vector to\_row\_vector(row\_vector)

89  template <typename T, int R, int C>

90  inline Matrix<T, 1, Dynamic>

91  to\_row\_vector(const Matrix<T, R, C>& matrix) {

92  return Matrix<T, 1, Dynamic>::Map(matrix.data(), matrix.rows()\*matrix.cols());

93  }

94

95  //row\_vector to\_row\_vector(real[])

96  template <typename T>

97  inline Matrix<T, 1, Dynamic>

98  to\_row\_vector(const vector<T> & vec) {

99  return Matrix<T, 1, Dynamic>::Map(vec.data(), vec.size());

100  }

101

102  //row\_vector to\_row\_vector(int[])

103  inline Matrix<double, 1, Dynamic>

104  to\_row\_vector(const vector<int> & vec) {

105  int C = vec.size();

106  Matrix<double, 1, Dynamic> result(C);

107  double\* datap = result.data();

108  for (int i=0; i < C; i++)

109  datap[i] = vec[i];

110  return result;

111  }

112

113  //real[,] to\_array\_2d(matrix)

114  template <typename T>

115  inline vector< vector<T> >

116  to\_array\_2d(const Matrix<T, Dynamic, Dynamic> & matrix) {

117  const T\* datap = matrix.data();

118  int C = matrix.cols();

119  int R = matrix.rows();

120  vector< vector<T> > result(R, vector<T>(C));

121  for (int i=0, ij=0; i < C; i++)

122  for (int j=0; j < R; j++, ij++)

123  result[j][i] = datap[ij];

124  return result;

125  }

126

127  //real[] to\_array\_1d(matrix)

128  //real[] to\_array\_1d(row\_vector)

129  //real[] to\_array\_1d(vector)

130  template <typename T, int R, int C>

131  inline vector<T> to\_array\_1d(const Matrix<T, R, C> & matrix) {

132  const T\* datap = matrix.data();

133  int size = matrix.size();

134  vector<T> result(size);

135  for (int i=0; i < size; i++)

136  result[i] = datap[i];

137  return result;

138  }

139

140  //real[] to\_array\_1d(...)

141  template <typename T>

142  inline vector<T>

143  to\_array\_1d(const vector<T> & x) {

144  return x;

145  }

146

147  //real[] to\_array\_1d(...)

148  template <typename T>

149  inline vector<typename scalar\_type<T>::type>

150  to\_array\_1d(const vector< vector<T> > & x) {

151  size\_t size1 = x.size();

152  size\_t size2 = 0;

153  if (size1 != 0)

154  size2 = x[0].size();

155  vector<T> y(size1\*size2);

156  for(size\_t i=0, ij=0; i < size1; i++)

157  for(size\_t j=0; j < size2; j++, ij++)

158  y[ij] = x[i][j];

159  return to\_array\_1d(y);

160  }

161

162  }

163 }

164 #endif

stan::math::to\_vector

Matrix< T, Dynamic, 1 > to\_vector(const Matrix< T, R, C > &matrix)

**Definition:** containers\_conversion.hpp:64

stan::math::to\_matrix

Matrix< T, Dynamic, Dynamic > to\_matrix(Matrix< T, R, C > matrix)

**Definition:** containers\_conversion.hpp:20

stan::math::to\_array\_2d

vector< vector< T > > to\_array\_2d(const Matrix< T, Dynamic, Dynamic > &matrix)

**Definition:** containers\_conversion.hpp:116

traits.hpp

stan::math::to\_row\_vector

Matrix< T, 1, Dynamic > to\_row\_vector(const Matrix< T, R, C > &matrix)

**Definition:** containers\_conversion.hpp:91

Eigen.hpp

stan::math::size

int size(const std::vector< T > &x)

**Definition:** size.hpp:11

stan::math::to\_array\_1d

vector< T > to\_array\_1d(const Matrix< T, R, C > &matrix)

**Definition:** containers\_conversion.hpp:131


---

[ Stan Home Page ]

*© 2011–2014,
Stan Development Team.*
